# Fine-resolution global maps of root biomass carbon colonized by arbuscular and ectomycorrhizal fungi

**DOI:** 10.1038/s41597-022-01913-2

**Published:** 2023-01-25

**Authors:** Milagros Barceló, Peter M. van Bodegom, Nadejda A. Soudzilovskaia

**Affiliations:** 1 Environmental Biology Department; Institute of Environmental Sciences, CML, Leiden University, Einsteinweg 2, 2333 CC Leiden, the Netherlands; 2 Centre for Environmental Sciences, Hasselt University, Martelarenlaan 42, 3500 Hasselt, Belgium

**Keywords:** Microbial ecology, Biogeography

## Abstract

Despite the recognized importance of mycorrhizal associations in ecosystem functioning, the actual abundance patterns of mycorrhizal fungi belowground are still unknown. This information is key for better quantification of mycorrhizal impacts on ecosystem processes and for incorporating mycorrhizal pathways into global biogeochemical models. Here we present the first high-resolution maps of fine root stocks colonized by arbuscular mycorrhizal (AM) and ectomycorrhizal (EcM) fungi (MgC ha−1). The maps were assembled by combining multiple open-source databases holding information on root biomass carbon, the proportion of AM and EcM tree biomass, plot-level relative abundance of plant species and intensity of AM and EcM root colonization. We calculated root-associated AM and EcM abundance in 881 spatial units, defined as the combination of ecoregions and land cover types across six continents. The highest AM abundances are observed in the (sub-)tropics, while the highest EcM abundances occur in the taiga regions. These maps serve as a basis for future research where continuous spatial estimates of root mycorrhizal stocks are needed.

## Background & summary

Mycorrhizal associations are key components of terrestrial ecosystems, influencing plant community composition^1,2^ and soil structure^3^ and biogeochemical fluxes^4–6^. However, these impacts have different magnitudes and directions depending on the mycorrhizal types involved. Arbuscular mycorrhiza (AM) and ectomycorrhiza (EcM) are the most taxonomically and geographically widespread mycorrhizal associations, being present in approximately 80% of the Earth’s plant species^7^ and are known to differ in their impacts^8,9^.

To quantify the impacts of different mycorrhizal types in ecosystem processes and to incorporate mycorrhizal pathways into global biogeochemical models we need information about the actual abundance of mycorrhizal associations in ecosystems. As a mutualistic association between a plant and a fungus, a comprehensive understanding of mycorrhizal abundance requires taking into account the biomass of each partner and the level of intimacy between them^9^.

Information about the relative abundance of distinct mycorrhizal host plants has been accumulating rapidly at regional^10–13^ and global scale^14,15^ as a tool to quantify the impact of mycorrhizal types on ecosystem functioning. By contrast, current knowledge about the abundance of mycorrhizal fungi belowground is still scarce, despite its direct impact on soil ecosystem functioning.

The belowground abundance of mycorrhizal fungi comprises the abundance of fungal mycelium in soil (extraradical mycelium) and plant roots (intraradical mycelium). As the assessment of the extraradical mycelium abundance is extremely difficult due to current methodological constraints^16^, this is poorly known at the regional and global scale. In contrast, the mycorrhizal abundance in plant roots is commonly reported in mycorrhizal literature^17^ as the proportion of root length colonized by AM fungi or root tips colonized by EcM fungi. Yet this information is typically provided for individual species and is rarely accompanied by data on the abundance of these species. While the intensity of root colonization of plant species quantifies the level of intimacy between plants and fungi, it still does not provide information about the actual abundance of intraradical mycelium of mycorrhizal fungi at the ecosystem level. For each mycorrhizal type, this parameter will ultimately depend on the total stock of fine roots capable to form given mycorrhizal associations (i.e. high colonization intensity but small root stocks will result in low total biomass of mycorrhizal fungi within plant roots).

Treseder *et al*.^18^ made the first quantification of total AM root stocks in different biomes. Yet, this analysis lacks spatial resolution and is based on very coarse estimations of AM plants’ abundance, root stocks and colonization intensity. Estimations of EcM root stocks are currently unavailable. In recent years, data on above and belowground plant abundance, and plant traits (including mycorrhizal traits) has been rapidly accumulating and compiled in open-access databases^17,19–21^. The release of these global high-quality data records allows making a big step in understanding patterns of mycorrhizal abundance belowground.

Here we present the first relatively fine-resolution map of AM and EcM abundance in roots (expressed as total carbon (C) in root biomass colonized by AM and EcM fungi). To assemble the map we combined data on root biomass C^20^, the proportion of AM and EcM tree biomass^14^,plot-level relative abundance of plant species^21^ and intensity of AM and EcM root colonization^17^. As a categorisation basis to create our maps, we calculated root-associated AM and EcM abundance in 881 spatial units, defined as the combination of ecoregions and land cover types across six continents. These units are relevant to the distribution of mycorrhizal host plants^14^ and enable higher spatial resolution than maps based on main biome classification. The new maps presented here (1) contribute to a better understanding of mycorrhizal global patterns, and allow examining drivers of these patterns, (2) help to identify the ecosystems that are more dependent on mycorrhizas and, consequently, more likely to be affected via changes in climatic conditions due to changes in mycorrhizal abundance, and (3) aid inferring mycorrhizal abundances in the soil matrix once the relationship between intraradical colonization and extraradical mycelium biomass^22^ has been confirmed at biome scale.

## Methods

**Fig. 1:**
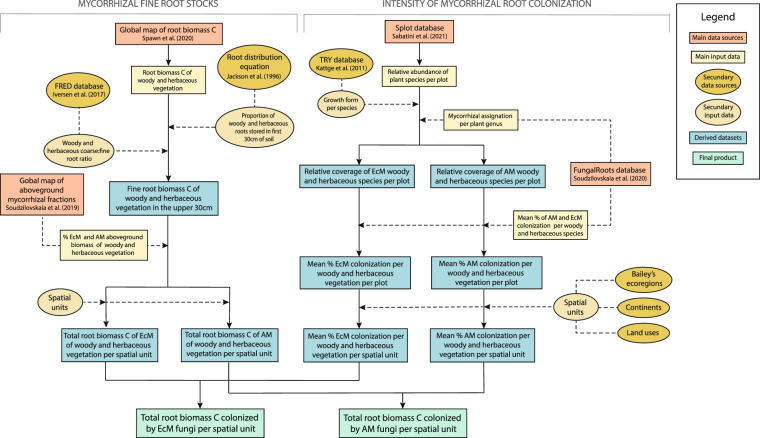
Workflow used to create maps of mycorrhizal fine root biomass carbon. The workflow consists of two main steps: (1) Estimation of total fine root stock capable to form mycorrhizal associations with AM and EcM fungi and (2) estimation of the proportion of fine roots colonized by AM and EcM fungi.

To calculate total root biomass C colonized by AM and EcM fungi, we developed a workflow that combines multiple publicly available datasets to ultimately link fine root stocks to mycorrhizal colonization estimates (Fig. 1). These estimates were individually derived for 881 different spatial units that were constructed by combining 28 different ecoregions, 15 land cover types and six continents. In a given spatial unit, the relationship between the proportion of AM- and EcM-plants aboveground biomass and the proportion of AM- and EcM-associated root biomass depends on the prevalence of distinct growth forms. Therefore, to increase the accuracy of our estimates, calculations were made separately for woody and herbaceous vegetation and combined in the final step and subsequently mapped. Below we detail the specific methodologies we followed within the workflow and the main assumptions and uncertainties associated.

### Definition of spatial units

As a basis for mapping mycorrhizal root abundances at a global scale, we defined spatial units based on a coarse division of Bailey’s ecoregions^23^ After removing regions of permanent ice and water bodies, we included 28 ecoregions defined according to differences in climatic regimes and elevation (deposited at Dryad-Table S1). A map of Bailey’s ecoregions was provided by the Oak Ridge National Laboratory Distributed Active Archive Center^24^ at 10 arcmin spatial resolution. Due to potential considerable differences in plant species identities, ecoregions that extended across multiple continents were split for each continent. The continent division was based upon the FAO Global Administrative Unit Layers (http://www.fao.org/geonetwork/srv/en/). Finally, each ecoregion-continent combination was further divided according to differences in land cover types using the 2015 Land Cover Initiative map developed by the European Space Agency at 300 m spatial resolution (https://www.esa-landcover-cci.org/). To ensure reliability, non-natural areas (croplands and urban areas), bare areas and water bodies were discarded (Table 1). In summary, a combination of 28 ecoregions, 15 land cover types and six continents were combined to define a total of 881 different spatial units (deposited at Dryad-Table S2). The use of ecoregion/land cover/continent combination provided a much greater resolution than using a traditional biome classification and allowed to account for human-driven transformations of vegetation, the latter based on the land cover data.

**Table 1:** List of land cover categories within the ESA CCI Land Cover dataset, used to assemble maps of mycorrhizal root biomass.

Original land cover class ID	Land cover class name
10	Cropland, rainfed
20	Cropland, irrigated or post-flooding
30	Mosaic cropland (>50%)/natural vegetation (tree, shrub,herbaceous cover) (<50%)
40	Mosaic natural vegetation (tree, shrub, herbaceous cover) (>50%)/cropland (<50%)
50	Tree cover, broadleaved, evergreen, closed to open (>15%)
60	Tree cover, broadleaved, deciduous, closed to open (>15%)
70	Tree cover, needleleaved, evergreen, closed to open (>15%–40%)
80	Tree cover, needleleaved, deciduous, closed to open (>15%)
90	Tree cover, mixed leaf type (broadleaved and needleleaved)
100	Mosaic tree and shrub (>50%)/herbaceous cover (<50%)
110	Mosaic herbaceous cover (>50%)/tree and shrub (<50%)
120	Shrubland
130	Grassland
140	Lichens and mosses
150	Sparse vegetation (tree, shrub, herbaceous cover)
160	Tree cover, flooded, fresh or brakish water
170	Tree cover, flooded, saline water
180	Shrub or herbaceous cover, flooded, fresh-saline or brakish water
190	Artificial surfaces and associated areas (Urban areas >50%)
200	Bare areas
201	Bare areas
202	Bare areas
210	Water bodies
220	Permanent snow and ice
0	No data (burn areas, clouds)

Land covers not included in the analisys are coloured. Source: ESA. CCI Land cover map 2015; https://www.esa-landcover-cci.org/.

### Mycorrhizal fine root stocks

#### Total root C stocks

Estimation of the total root C stock in each of the spatial units was obtained from the harmonized belowground biomass C density maps of Spawn *et al*.^20^. These maps are based on continental-to-global scale remote sensing data of aboveground biomass C density and land cover-specific root-to-shoot relationships to generate matching belowground biomass C maps. This product is the best up-to-date estimation of live root stock available. For subsequent steps in our workflow, we distinguished woody and herbaceous belowground biomass C as provided by Spawn *et al*.^20^. As the tundra belowground biomass C map was provided without growth form distinction, it was assessed following a slightly different workflow (see Section 2.2.3 for more details). To match the resolution of other input maps in the workflow, all three belowground biomass C maps were scaled up from the original spatial resolution of 10-arc seconds (approximately 300 m at the equator) to 10 arc‐minutes resolution (approximately 18.5 km at the equator) using the mean location of the raster cells as aggregation criterion.

As the root biomass C maps do not distinguish between fine and coarse roots and mycorrhizal fungi colonize only the fine fractions of the roots, we considered the fine root fraction to be 88,5% and 14,1% of the total root biomass for herbaceous and woody plants, respectively. These constants represent the mean value of coarse/fine root mass ratios of herbaceous and woody plants provided by the Fine-Root Ecology Database (FRED) (https://roots.ornl.gov/)^25^ (deposited at Dryad-Table S3). Due to the non-normality of coarse/fine root mass ratios, mean values were obtained from log-transformed data and then back-transformed for inclusion into the workflow.

Finally, the belowground biomass C maps consider the whole root system, but mycorrhizal colonization occurs mainly in the upper 30 cm of the soil^18^. Therefore, we estimated the total fine root stocks in the upper 30 cm by applying the asymptotic equation of vertical root distribution developed by Gale & Grigal^26^:

$$y=1-{\beta }^{d}$$

where *y* is the cumulative root fraction from the soil surface to depth *d* (cm), and β is the fitted coefficient of extension. β values of trees (β = 0.970), shrubs (β = 0.978) and herbs (β = 0.952) were obtained from Jackson *et al*.^27^. A mean value was then calculated for trees and shrubs to obtain a woody vegetation β value of 0.974. As a result, we estimated that 54.6% of the total live root of woody vegetation and 77.1% of herbaceous vegetation is stored in the upper 30 cm of the soil. In combination, this allowed deriving fine root C stocks in the upper 30 cm of woody and herbaceous vegetation.

#### The proportion of root stocks colonized by AM and EcM

The proportion of root stock that forms associations with AM or EcM fungi was obtained from the global maps of aboveground biomass distribution of dominant mycorrhizal types published by Soudzilovskaia *et al*.^14^. These maps provide the relative abundance of EcM and AM plants based on information about the biomass of grass, shrub and tree vegetation at 10arcmin resolution. To match with belowground root woody plants biomass data, proportions of AM trees and shrubs underlying the maps of Soudzilovskaia *et al*.^14^ were summed up to obtain the proportion of AM woody vegetation. The same was done for EcM trees and shrubs.

**Fig. 2:**
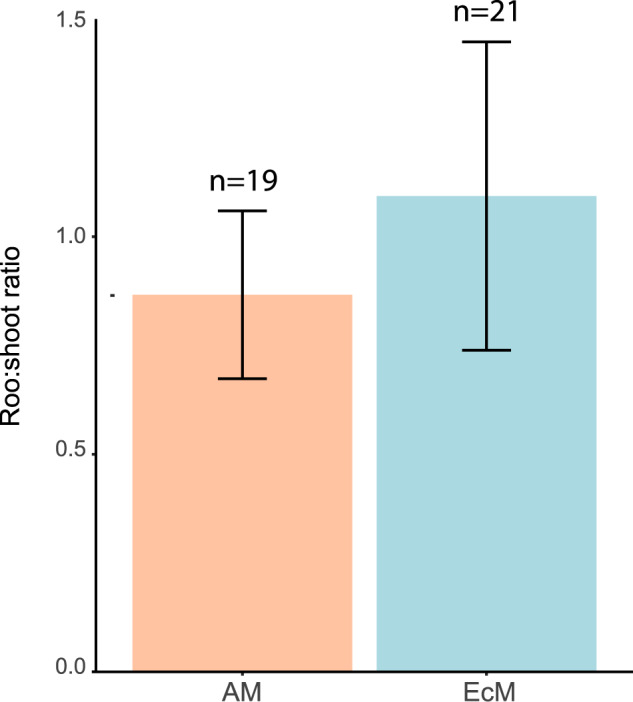
Mean and standar error of root to shoot ratios of AM and EcM woody plant species.

Our calculations are subjected to the main assumption that, within each growth form, the proportion of aboveground biomass associated with AM and EcM fungi reflects the proportional association of AM and EM fungi to belowground biomass. We tested whether root:shoot ratios were significantly different between AM and EcM woody plants (the number of EcM herbaceous plants is extremely small^17^). Genera were linked to growth form based on the TRY database (https://www.try-db.org/)^19^ and the mycorrhizal type association based on the FungalRoots database^17^. Subsequently, it was tested whether root:shoot ratios of genera from the TRY database (https://www.try-db.org/)^19^ were significantly different for AM vs EcM woody plants. No statistically significant differences (ANOVA-tests p-value = 0.595) were found (Fig. 2).

#### Estimation of mycorrhizal fine root stocks

**Fig. 3:**
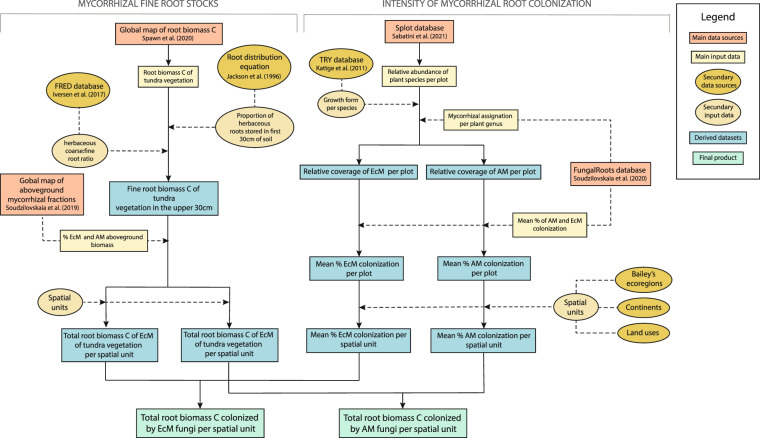
Workflow used to create mycorrhizal fine root biomass C maps specific for tundra areas.

We calculated the total biomass C of fine roots that can potentially be colonized by AM or EcM fungi by multiplying the total woody and herbaceous fine root C biomass in the upper 30 cm of the soil by the proportion of AM and EcM of woody and herbaceous vegetation. In the case of tundra vegetation, fine root C stocks were multiplied by the relative abundance of AM and EcM vegetation without distinction of growth forms (for simplicity, this path was not included in Fig. 1, but can be seen in Fig. 3. As tundra vegetation consists mainly of herbs and small shrubs, the distinction between woody and herbaceous vegetation is not essential in this case.

**Fig. 4:**
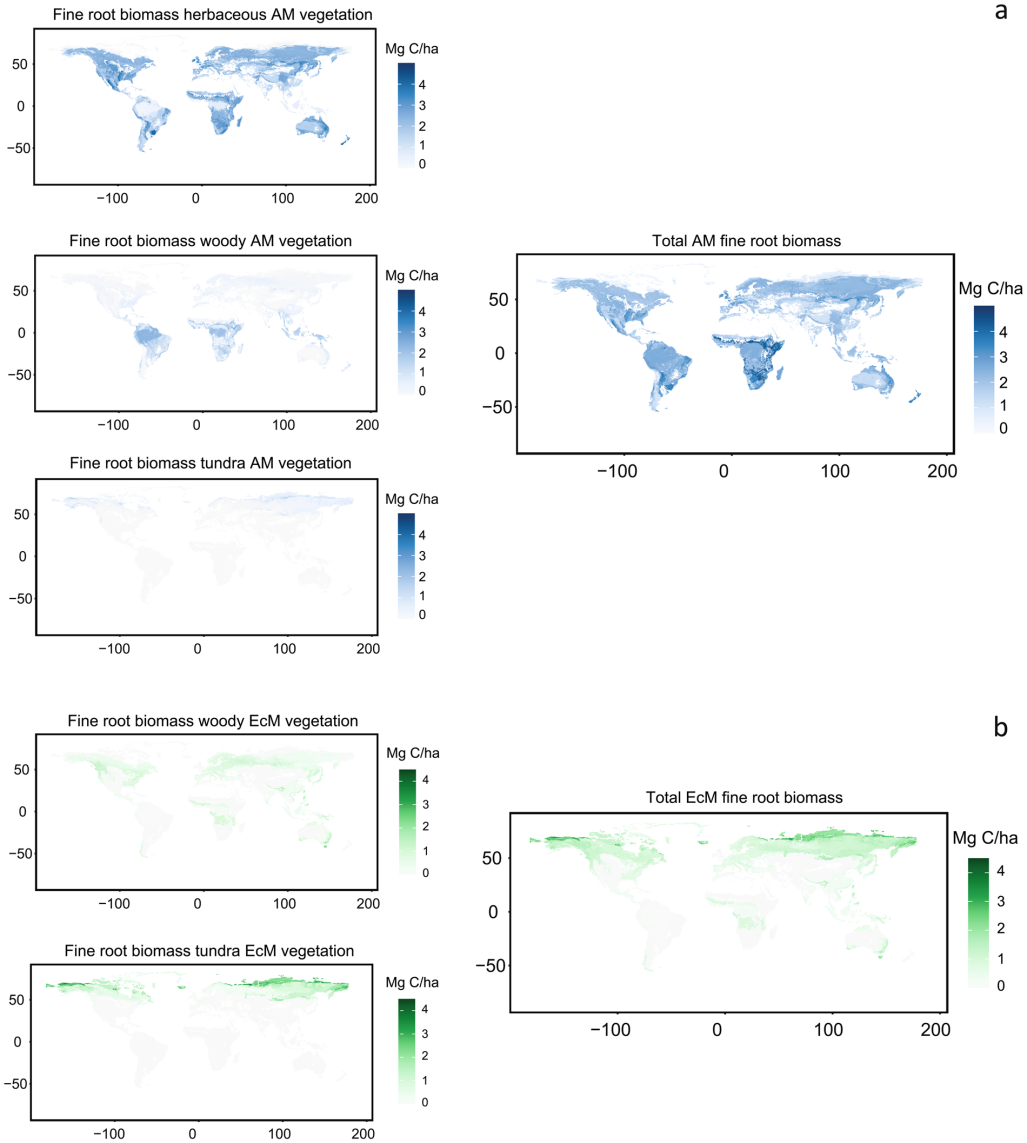
Fine root biomass stocks capable to form association with AM (**a**) and EcM (**b**) fungi for woody, herbaceous and tundra vegetation. Final AM and EcM stock result from the sum of the growth form individual maps. There were no records of fine root biomass of EcM herbaceous vegetation.

Finally, we obtained the mean value of mycorrhiza growth form fine root C stocks in each of the defined spatial units. These resulted in six independent estimations: AM woody, AM herbaceous, EcM woody, EcM herbaceous, AM tundra and EcM tundra total fine root biomass C (Fig. 4).

### The intensity of root colonization by mycorrhizal fungi

#### Colonization database

**Fig. 5:**
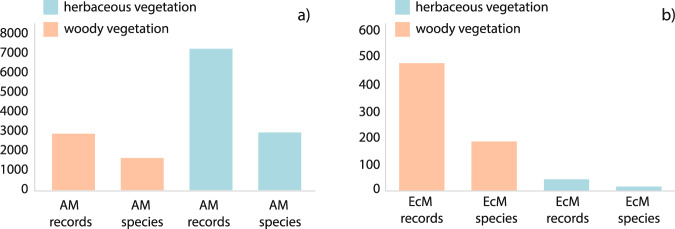
Number of AM (**a**) and EcM (**b**) herbaceous and woody plant species and total observations obtained from FungalRoot database.

The FungalRoot database is the largest up-to-date compilation of intensity of root colonization data, providing 36303 species observations for 14870 plant species. Colonization data was filtered to remove occurrences from non-natural conditions (i.e., from plantations, nurseries, greenhouses, pots, etc.) and data collected outside growing seasons. Records without explicit information about habitat naturalness and growing season were maintained as colonization intensity is generally recorded in the growing season of natural habitats. When the intensity of colonization occurrences was expressed in categorical levels, they were converted to percentages following the transformation methods stated in the original publications. Finally, plant species were distinguished between woody and herbaceous species using the publicly available data from TRY (https://www.trydb.org/)^19^. As a result, 9905 AM colonization observations of 4494 species and 521 EcM colonization observations of 201 species were used for the final calculations (Fig. 5).

The use of the mean of mycorrhizal colonization intensity per plant species is based on two main assumptions:

1. **The intensity of root colonization is a plant trait**: It is known that the intensity of mycorrhizal infections of a given plant species varies under different climatic and soil conditions^28,29^, plant age^30^ and the identity of colonizing fungal species^31^. However, Soudzilovskaia *et al*.^9^ showed that under natural growth conditions the intraspecific variation of root mycorrhizal colonization is lower than interspecific variation, and is within the range of variations in other plant eco-physiological traits. Moreover, recent literature reported a positive correlation between root morphological traits and mycorrhizal colonization, with a strong phylogenetic signature of these correlations^32,33^. These findings provide support for the use of mycorrhizal root colonization of plants grown in natural conditions as a species-specific trait.
2. **The percentage of root length or root tips colonized can be translated to the percentage of biomass colonized:** intensity of root colonization is generally expressed as the proportion of root length colonized by AM fungi or proportion of root tips colonized by EcM fungi (as EcM infection is restricted to fine root tips). Coupling this data with total root biomass C stocks requires assuming that the proportion of root length or proportion of root tips colonized is equivalent to the proportion of root biomass colonized. While for AM colonization this equivalence can be straightforward, EcM colonization can be more problematic as the number of root tips varies between tree species. However, given that root tips represent the terminal ends of a root network^34^, the proportion of root tips colonized by EcM fungi can be seen as a measurement of mycorrhizal infection of the root system and translated to biomass independently of the number of root tips of each individual. Yet, it is important to stress that estimations of fine root biomass colonized by AM and EcM as provided in this paper might not be directly comparable.

#### sPlot database

The sPlotOpen database^21^ holds information about the relative abundance of vascular plant species in 95104 different vegetation plots spanning 114 countries. In addition, sPlotOpen provides three partially overlapping resampled subset of 50000 plots each that has been geographically and environmentally balanced to cover the highest plant species variability while avoiding rare communities. From these three available subsets, we selected the one that maximizes the number of spatial units that have at least one vegetation plot. We further checked if any empty spatial unit could be filled by including sPlot data from other resampling subsets.

Plant species in the selected subset were classified as AM and EcM according to genus-based mycorrhizal types assignments, provided in the FungalRoot database^17^. Plant species that could not be assigned to any mycorrhizal type were excluded. Facultative AM species were not distinguished from obligated AM species, and all were considered AM species. The relative abundance of species with dual colonization was treated as 50% AM and 50% ECM. Plant species were further classified into woody and herbaceous species using the TRY database.

#### Estimation of the intensity of mycorrhizal colonization

The percentage of AM and EcM root biomass colonized per plant species was spatially upscaled by inferring the relative abundance of AM and EcM plant species in each plot. For each mycorrhizal-growth form and each vegetation plot, the relative abundance of plant species was determined to include only the plant species for which information on the intensity of root colonization was available. Then, a weighted mean intensity of colonization per mycorrhizal-growth form was calculated according to the relative abundance of the species featuring that mycorrhizal-growth form in the vegetation plot. Lastly, the final intensity of colonization per spatial unit was calculated by taking the mean value of colonization across all plots within that spatial unit. These calculations are based on 38127 vegetation plots that hold colonization information, spanning 384 spatial units.

The use of vegetation plots as the main entity to estimate the relative abundance of AM and EcM plant species in each spatial unit assumes that the plant species occurrences and their relative abundances in the selected plots are representative of the total spatial unit. This is likely to be true for spatial units that are represented by a high number of plots. However, in those spatial units where the number of plots is low, certain vegetation types or plant species may be misrepresented. We addressed this issue in our uncertainty analysis. Details are provided in the Quality index maps section.

### Final calculation and maps assembly

The fraction of total fine root C stocks that is colonized by AM and EcM fungi was estimated by multiplying fine root C stocks by the mean root colonization intensity in each spatial unit. This calculation was made separately for tundra, woody and herbaceous vegetation.

To generate raster maps based on the resulting AM and EcM fine root biomass C data, we first created a 10 arcmin raster map of the spatial units. To do this, we overlaid the raster map of Bailey ecoregions (10 arcmin resolution)^24^, the raster of ESA CCI land cover data at 300 m resolution aggregated to 10 arcmin using a nearest neighbour approach (https://www.esa-landcover-cci.org/) and the FAO polygon map of continents (http://www.fao.org/geonetwork/srv/en/), rasterized at 10 arcmin. Finally, we assigned to each pixel the corresponding biomass of fine root colonized by mycorrhiza, considering the prevailing spatial unit. Those spatial units that remained empty due to lack of vegetation plots or colonization data were filled with the mean value of the ecoregion x continent combination.

### Quality index maps

As our workflow comprises many different data sources and the extracted data acts in distinct hierarchical levels (i.e plant species, plots or spatial unit level), providing a unified uncertainty estimation for our maps is particularly challenging. Estimates of mycorrhizal fine root C stocks are related mainly to belowground biomass C density maps and mycorrhizal aboveground biomass maps, which have associated uncertainties maps provided by the original publications. In contrast, estimates of the intensity of root colonization in each spatial unit have been associated with three main sources of uncertainties:

1. **The number of observations in the FungalRoot database**. The mean species-level intensity of mycorrhizal colonization in the vegetation plots has been associated with a number of independent observations of root colonization for each plant species. We calculated the mean number of observations of each plant species for each of the vegetation plots and, subsequently the mean number of observations (per plant species) from all vegetation plots in each spatial unit. These spatial unit averaged number of observations ranged from 1 to 14 in AM and from 1 to 26 in EcM. A higher number of observations would indicate that the intraspecific variation in the intensity of colonization is better captured and, therefore, the species-specific colonization estimates are more robust.
2. **The relative plant coverage that was associated with colonization data**. From the selected vegetation plots, only a certain proportion of plant species could be associated with the intensity of colonization data in FungalRoot database. The relative abundance of the plant species with colonization data was summed up in each vegetation plot. Then, we calculated the average values for each spatial unit. Mean abundance values ranged from 0.3 to 100% in both AM and EcM spatial units. A high number indicates that the dominant plant species of the vegetation plots have colonization data associated and, consequently, the community-averaged intensity of colonization estimates are more robust.
3. **The number of vegetation plots in each spatial unit**. Each of the spatial units differs in the number of plots used to calculate the mean intensity of colonization, ranging from 1 to 1583 and from 1 to 768 plots in AM and EcM estimations, respectively. A higher number of plots is associated with a better representation of the vegetation variability in the spatial units, although this will ultimately depend on plot size and intrinsic heterogeneity (i.e., a big but homogeneous spatial unit may need fewer vegetation plots for a good representation than a small but very heterogeneous spatial unit).

We provide independent quality index maps of the spatial unit average of these three sources of uncertainty. These quality index maps can be used to locate areas where our estimates have higher or lower robustness.

## Data Records

**Fig. 6:**
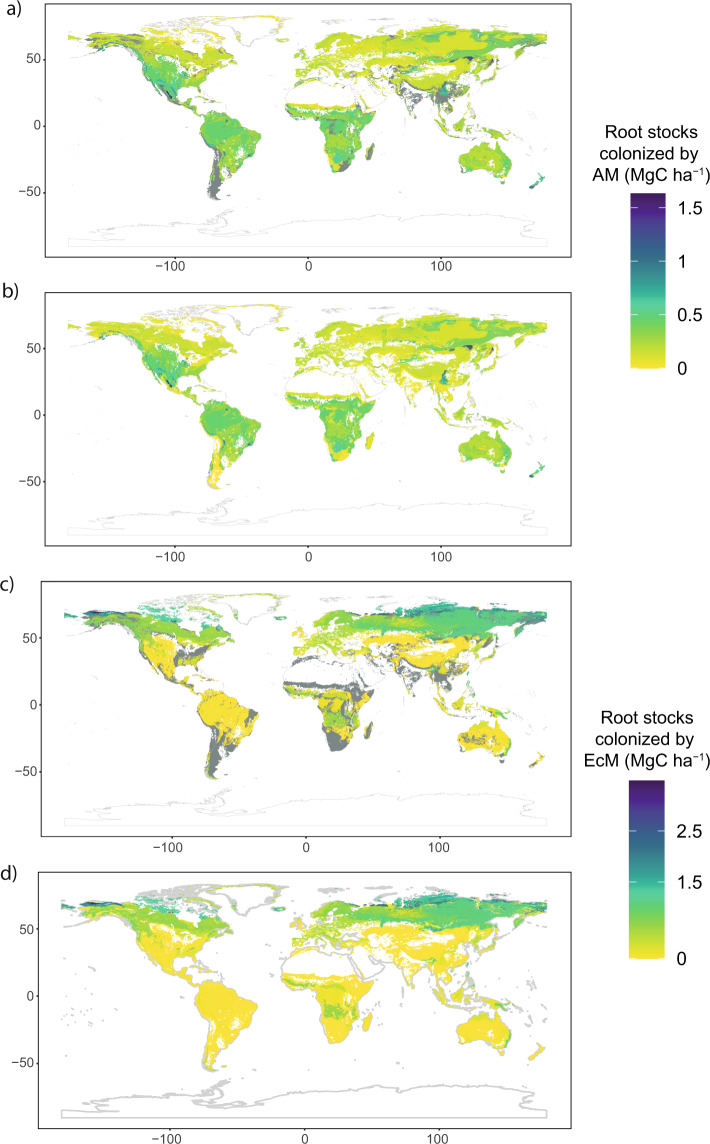
Maps of fine root biomass C colonized by AM and EcM (MgC ha-1) including in grey spatial units with an absence of data (**a**,**c**) and maps with empty spatial units covered (**b**,**d**).

**Fig. 7:**
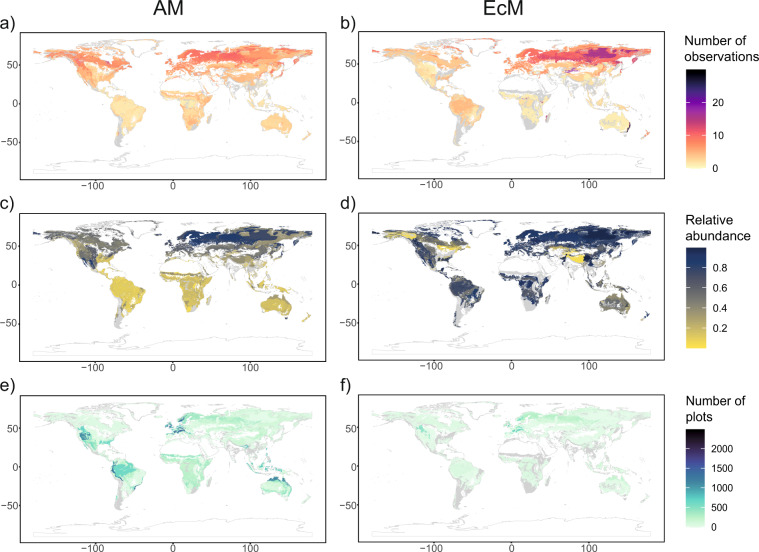
Quality index maps associated with AM and EcM total root biomass C: (**a**,**b**) The average number of occurrences in the colonization database per species in each spatial unit, (**c**,**d**) mean relative abundance of species with colonization data per spatial unit (**e**,**f**). The number of plots included in each spatial unit.

Maps on AM and EcM root biomass C (Fig. 6), as well as their associated quality index maps (Fig. 7), are available as individual raster files in tiff format. In addition, we provide herbaceous, woody and tundra AM and EcM root biomass C raster files used to construct the main product. Finally, we provide raster files of total root stocks capable to form associations with AM and EcM fungi and the intensity of AM and EcM root colonization as intermediate products. Coordinates of all raster layers are expressed in longitude/latitude relative to the WGS84 system and the spatial resolution is 10 arc-min. These files are available through the public repository Dryad^35^ (10.5061/dryad.866t1g1tt). Table 2 provides a summary of the available files.

**Table 2:** Description of the available raster layers.

Raster name	Description	Units
AM_roots_colonized	Fine root biomass carbon stocks associated with AM fungi	MgC ha^−1^
EcM_roots_colonized	Fine root biomass carbon stocks associated with EcM fungi	MgC ha^−1^
AM_herbs_roots_colonized	Herbaceous fine root biomass carbon stocks associated with AM fungi	MgC ha^−1^
AM_woody_roots_colonized	Woody fine root biomass carbon stocks associated with AM fungi	MgC ha^−1^
EcM_woody_roots_colonized	Woody fine root biomass carbon stocks associated with EcM fungi	MgC ha^−1^
AM_tundra_roots_colonized	Tundra fine root biomass carbon stocks associated with AM fungi	MgC ha^−1^
EcM_tundra_roots_colonized	Tundra fine root biomass carbon stocks associated with EcM fungi	MgC ha^−1^
AM_occurrences_colonization	Averaged number of occurrences in the colonization database of AM species per each spatial unit	Number of occurrences
EcM_occurrences_colonization	Averaged number of occurrences in the colonization database of EcM species per spatial unit	Number of occurrences
AM_rel.abundance_colonization	Mean relative abundance of AM species with colonization data per spatial unit	%
EcM_rel.abundance_colonization	Mean relative abundance of EcM species with colonization data per spatial unit	%
AM_plots	The number of plots included each spatial unit for AM calculation	Number of sPlots
EcM_plots	The number of plots included each spatial unit for EcM calculation	Number of sPlots
AM_roots	Fine root biomass C stocks that are capable to form associations with AM fungi	MgC ha^−1^
EcM_roots	Fine root biomass C stocks that are capable to form associations with EcM fungi	MgC ha^−1^
AM_intensity_colonization	The intensity of AM root colonization	%
EcM_intensity_colonization	The intensity of EcM root colonization	%
Spatial_units	Table describing each of the 881 defined spatial units	—
Bailey_ecoregions	Table describing the codes and division of Bailey ecoregions	—
Coarse_vs_fine_roots	Table including coarse root/fine root mass ratio records used to calculate growth-form means	—

## Technical Validation

Our maps are based on the combination of other maps and databases that have been previously curated and validated by the original publishers and represent the most recent and high-quality open-access information available. Through our workflow, we ensured that the introduction of errors is minimized by avoiding data that can generate biased results. Due to the lack of large-scale empirical data on mycorrhizal fungal biomass in roots or any other similar estimates, it is not feasible to directly assess the validity of our final estimates.

In order to validate our maps, in view of this complications, we opted for evaluating the two main sections of our workflow independently. The values of fine root stocks are directly calculated by multiplying the data from two main global maps (root biomass and mycorrhizal aboveground biomass fractions), which have been previously validated and have associated uncertainty maps available in the original publications. Therefore, we consider that providing an additional validation with independent data, which is currently not available, is not essential in this case. In contrast, the estimation of the intensity of mycorrhizal root colonization is more complex, as it combines multiple datasets with information in different hierarchical levels and there is no associated uncertainty estimation. Therefore, for this part of data a validation effort becomes crucial to generate confidence in our estimates. For such purpose, we compared our estimates with the global map of the intensity of AM and EcM intensity of root colonization provided by Soudzilovskaia *et al*.^36^. That map presents colonization of roots present in an ecosystem (i.e in contrast to the product presented here not accounting for root biomass) as predicted by climate and soil conditions. For better consistency during the validation process, we excluded from the comparison data points of the dataset of Soudzilovskaia *et al*.^36^ associated with a standard error higher than 20 percent units, i.e. those were uncertainty in the data on percent of root length colonized, exceeds the value of 20%.

**Fig. 8:**
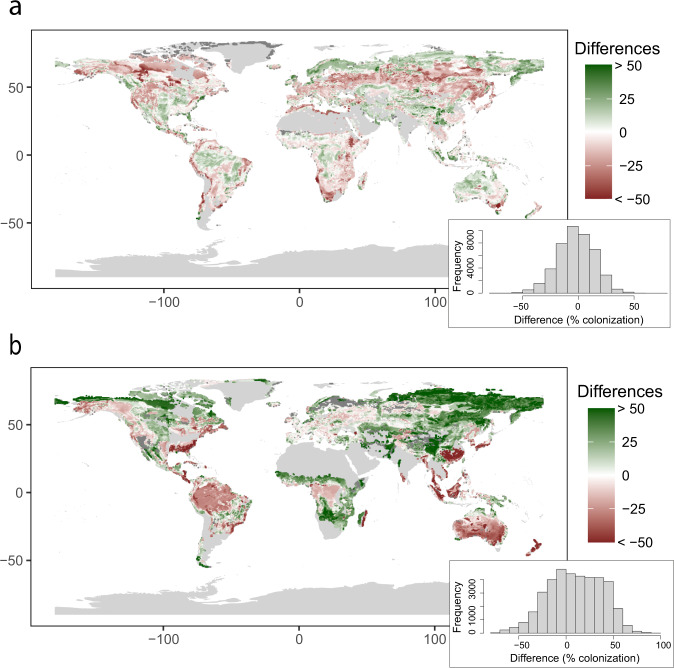
Spatial representation and related histograms of differences between the percentage of AM (**a**) and EcM (**b**) root colonization between our estimates and the global maps on the intensity of root colonization of Soudzilovskaia *et al*.^36^.

In general, our AM intensity of colonization estimates do not show important deviations with respect to Soudzilovskaia *et al*.^36^, with most of the data differing by less than 20% of colonization and not showing any clear spatial pattern (Fig. 8a). In the case of EcM, discrepancies are higher (Fig. 8b), being more evident in the northeast area of Asia and tundra zones, where our estimates show approximately 50% more root tips colonized by EcM than those in the comparing map. It is important to notice that the areas with major discrepancies correspond to those where extrapolations of Soudzilovskaia *et al*.^36^ were based on a very low number of data points. Given that our estimates in those areas are based on a higher number of vegetation plots and that in boreal forests EcM trees are known to be intensively colonized^37^, we suggest that our predictions improve those from Soudzilovskaia *et al*.^36^.

## Usage Notes

The final raster layers produced here are suitable for a broad variety of applications where continuous spatial estimates of root mycorrhizal stocks are needed. These include assessments of mycorrhizal impacts on ecosystem processes, global analyses of mycorrhizae distribution and its environmental drivers or inclusion of mycorrhizal pathways on global biogeochemical models. Additionally, the release of woody and herbaceous mycorrhizal root maps allows applications focused on specific growth forms.

Our maps are intended for continental or large scales applications. Users interested in regional or local assessments must be especially aware of the limitation and uncertainties associated with the databases used to generate the maps in the region of interest. This is particularly relevant for tropical areas that concentrate higher uncertainties and lack of data, making our estimations less robust.

Finally, it is important to note that, while our estimates can be seen as a proxy for mycorrhizal abundance within plant roots, they cannot be directly translated to intraradical (beyond roots) fungal biomass. Equations that transform root colonization into mycorrhizal fungal biomass^38^ are available. However, as these formulas involve root length colonization instead of root biomass colonization, they cannot be directly applied to our estimations.

## Data Availability

All mycorrhizal root biomass maps and their associated products were created in R statistical computing environment^39^. The code consists of five main interconnected scripts that are stored in Github repository (https://github.com/milimdp/Fine-resolution-global-maps-of-root-biomass-C-colonized-by-AM-and-EcM-fungi-). 1. “Spatial_units_map”: Creates raster map of spatial units 2. “Colonization_spatial_units”: Calculates the mean percentage of colonization per spatial unit 3. “Mycorrhizal_roots_biomass_stapial_unit”: Calculates mean mycorrhizal root biomass per spatial unit 4. “Final_maps”: Creates final raster maps 5. “Quality_index”: Calculates quality index per spatial unit and creates maps
